# A Global Analysis of Associations between Fine Particle Air Pollution and Cardiovascular Risk Factors: Feasibility Study on Data Linkage

**DOI:** 10.5334/gh.877

**Published:** 2020-08-06

**Authors:** Min Zhao, Gerard Hoek, Maciej Strak, Diederick E. Grobbee, Ian Graham, Kerstin Klipstein-Grobusch, Ilonca Vaartjes

**Affiliations:** 1 Julius Global Health, Julius Center for Health Sciences and Primary Care, University Medical Center Utrecht, Utrecht University, Utrecht, NL; 2 Institute for Risk Assessment Sciences, Utrecht, Utrecht University, Utrecht, NL; 3 Global Geo and Health Data Center, Utrecht University, Utrecht, NL; 4 Trinity College Dublin, Dublin, IE; 5 Division of Epidemiology and Biostatistics, School of Public Health, Faculty of Health Sciences, University of the Witwatersrand, Johannesburg, ZA

**Keywords:** air pollution, environmental health, cardiovascular disease, risk factors, feasibility, data linkage

## Abstract

**Background:**

This paper presents a feasibility study of data linkage between global air pollution data and clinical medical data to assess the associations of PM_2.5_ with cardiovascular risk factors.

**Methods:**

Cardiovascular risk factor data were obtained from the SUrvey of Risk Factors (SURF) for coronary heart disease (CHD) patients from 10 countries in Europe, Asia, and the Middle-East. Annual average PM_2.5_ concentrations were estimated using recent global WHO PM_2.5_ maps combining satellite and surface monitoring data for the location of the 71 participating centers. Associations of PM_2.5_ with risk factors were assessed by mixed-effect generalized estimation equation models adjusted by sex, age, exercise, body mass index, and smoking. In the final model there was further adjustment for country.

**Results:**

Linkage between cardiovascular risk factor data and PM_2.5_ via the postal address of participating hospitals was shown to be feasible, however with several limitations noted.

Eight thousand three hundred and ninety two patients (30% women) were included. Globally, an increase of 10 μg/m^3^ in PM_2.5_ was significantly associated with decreased BP and increased glucose. After controlling for country, an increase of 10 μg/m^3^ in PM_2.5_ was associated with decreased BP and increased LDL (SBP: –0.45 mmHg [95% CI: –0.85, –0.06]; DBP: –0.47 mmHg [–0.73, –0.20]; LDL: 0.04 mmol/L [0.01, 0.08]). The association with glucose attenuated (0.08 mmol/L [–0.23, 0.16]).

**Conclusion:**

It is feasible to link PM_2.5_ and cardiovascular risk factors but it is still challenging to interpret these observed associations due to unavailability of potential confounders. After country adjustment, PM_2.5_ was associated with small increases in LDL and small decreases in BP.

**Highlights:**

- There are limited studies on the association between air pollution and cardiovascular risk factors for patients with established coronary heart disease in low- and middle-income countries;
- Data linkage is an efficient and cost-effective method to maximize the use of existing data to investigate more health related research questions;
- It is feasible to determine global associations of air pollution and cardiovascular risk factors by data linkage but it is still challenging in terms of interpretation.

## Background

Cardiovascular disease (CVD) remains one of the leading causes of death worldwide with 18 million deaths in 2016 [1]. Traditionally, evidence based guidelines and daily practice on secondary prevention of CVD have focused on modifiable risk factor management [2,3]. Several recent epidemiological studies have suggested air pollution could also be associated with CVD risks [4,5,6,7]. The number of studies investigating the association between PM_2.5_ and modifiable cardiovascular risk factors is scarce [8,9,10,11,12,13,14]. These studies have predominantly been conducted in Western countries with rather low levels of PM_2.5_ concentrations [8,9,10]. In contrast, low- and middle-income countries, for which have limited data on the association of PM_2.5_ and risk factors, show much higher PM_2.5_ concentrations [15]. Existing evidence on the role of environmental exposure on cardiovascular risk factors may however not be generalizable to these settings since the chemical composition and characteristics of PM_2.5_ may differ significantly from those in Western countries [15]. This, together with a rapid increase of CVD prevalence in low- and middle-income countries, stresses the importance of a better understanding of global associations of PM_2.5_ with cardiovascular risk factors.

Conducting targeted studies on the association between PM_2.5_ and cardiovascular risk factors on a global scale is challenging. Data linkage is an efficient and cost-effective method to maximize the use of existing data for more health related research questions [16,17]. Current study aims to assess the feasibility of linking global air pollution data with the cardiovascular risk factors data collected from an international audit to establish the technical and scientific possibilities on data linkage. This study also aims to investigate the potential association between PM_2.5_ and cardiovascular risk factors (Blood pressure <BP>, total cholesterol <TC>, low-density lipoprotein cholesterol <LDL>, high-density lipoprotein cholesterol <HDL>, and glucose) among patients with established coronary heart disease (CHD) in Europe, Asia, and the Middle East.

## Methods

### Study population and outcomes

We used cardiovascular risk factors from the SUrvey of Risk Factors (SURF). Details have been reported previously [18,19]. Briefly, SURF was a clinical audit carried during routine cardiology visit in ten countries among three regions, including Europe (Croatia, Denmark, Ireland, Italy, Northern Ireland, Romania, Russia), Asia (Mainland of China and Taiwan), and Middle East (Saudi Arabia). Within each center, patients aged ≥18 years with a clinical diagnosis of CHD (coronary artery bypass surgery <CABG>, percutaneous coronary intervention <PCI>, acute coronary syndromes <ACS> or stable angina) were recruited between 2012 and 2013. Data on patient demographics (age, sex, and center location), lifestyles (smoking status and physical activity), physical and laboratory measurements (body anthropometry, BP, TC, LDL, HDL, and glucose), and medications were collected by trained research staffs using one-page data collection. BP, lipids, and glucose were measured according to local national guidelines and retrieved directly from medical records.

### Air pollution data

We extracted annual average PM_2.5_ concentrations from the World Health Organization (WHO) database (http://www.who.int/phe/health_topics/outdoorair/databases/modelled-estimates/en/). The database provides estimates of annual average concentration of PM_2.5_ at a spatial resolution of 0.1° × 0.1°, which is approximately 11 × 11 km at the equator globally. Due to data availability, we used annual average of the year 2014. The estimates are based on the recently developed Data Integration Model for Air Quality [20]. The model estimates PM_2.5_ using satellite retrievals of aerosol optical depth, chemical transport models, population estimates, topography and ground measurements from 6003 stations worldwide. A Bayesian hierarchical model is used to integrate these information sources [20]. The major advantage of the model is that estimates are available from a consistent method globally, as opposed to ground measurements, which are concentrated in limited regions of the world.

We additionally collected data in 2013 for European centers from countries that report measurements data to the European Environment Agency using the Airbase database (https://www.eea.europa.eu/data-and-maps/data/airbase-the-european-air-quality-database-7). For the 17 districts in the city of Beijing we also obtained online PM_2.5_ data from the Beijing Municipal Environmental Protection Bureau for the year 2013.

### Linkage of the data sources

The postal address of each clinic was transformed into geographical coordinates-the latitude, longitude coordinate system (5 digits)-using Google Earth. We first linked PM_2.5_ data from the background monitoring stations in the town itself. If no station was available, we estimated PM_2.5_ from the more frequently measured pollutant PM_10_ if available or used the average of the nearest two background stations if PM_10_ was also not available. We used country-specific ratios from EEA database to convert PM_10_ into PM_2.5_ fractions if available. If not available, we used PM_2.5_/PM_10_ = 0.60 from a large European project or a generic PM_2.5_/PM_10_ ratio of 0.60 from a large European project if no country-specific estimates were available [21]. For a small town, we used regional stations and for a large city urban stations.

### Statistical analyses

The associations of cardiovascular risk factors with an increase of 10 μg/m^3^ in PM_2.5_ were assessed by adjusted mixed-effect generalized estimation equation models. Patient’s characteristics and lifestyles varied country by country and thus, we included all available potential confounding factors related to both cardiovascular risk factors and PM_2.5_, including sex, age, and individual risk factors (physical activity <low, moderate, vigorous>, smoking status <current smoker, ex-smoker, never>, and body mass index <BMI>) [22]. All patients with established CHD were expected to be on cardiovascular medications to prevent the recurrence of cardiac event irrespective of geographical areas. Thus, cardiovascular medications were not included as a confounder. We further adjusted for country as a fixed covariate as a proxy for potential unknown and known confounders for which we did not have individual information. All outcomes were also nested within center (the random effect) to allow for clustering within centers.

Imputed data were analyzed in the primary analysis. There were about less than 4% missing data for all variables (Appendix Table A). Ten datasets were imputed for missing data with multivariate imputation by chained equations (MICE package in R) [23]. Briefly, MICE predicts missing data by iteratively optimizing a series of regression models using other potentially predictive variables such as basic demographics and geographic area. The continuous variables including height, weight, BP, TC, LDL, HDL, and glucose were imputed by predictive mean matching and the categorical data including smoking status and physical activity were imputed with logistic regression.

Because of uncertainty of the shape of the concentration response function at high concentrations, we performed sensitivity analyses excluding the two countries with the highest PM_2.5_ levels (China and Saudi Arabia) (Appendix Figure A and B). We further analyzed associations of PM_2.5_ retrieved from the Airbase for European countries and the database from the Beijing Municipal Environmental Protection Bureau for China with the same statistical strategy.

Statistical analyses were performed by using ‘MICE’ and ‘GEEPACK’ packages in R [23,24]. All tests were two tailed with statistical significance assumed at the 0.05 level.

## Results

We first describe the collected data and associations between air pollution and cardiovascular risk factors and then summarize the potential limitations of using existing audit data.

### Baseline characteristics

A total of 8392 SURF patients were included. The mean age of all patients was 64.9 years; 29.6% were women; 16% reported current smoker (Table 1). The average overall systolic blood pressure (SBP), diastolic blood pressure (DBP), TC, LDL, HDL, and glucose were 131.1 mmHg, 75.8 mmHg, 4.2 mmol/L, 2.4 mmol/L, 1.1 mmol/L, and 7.5 mmol/L, respectively. The average PM_2.5_ level from WHO database was 38.1 μg/m^3^, ranging from 10.1 μg/m^3^ in Ireland to 92.7 μg/m^3^ in Saudi Arabia. Appendix Figure B illustrates the large variation of individual outcome variables, especially within countries.

**Table 1:** Description of patient characteristics, cardiovascular risk factors, and air pollutants.

	Overall	Europe	Croatia	Denmark	Ireland	Italy	NI	Russia	Romania	KSA	Taiwan	Beijing

No.	8392	5001	1223	300	1716	771	159	463	369	1509	732	1150
No. of Centre	71	51	8	1	11	14	2	8	7	4	4	12
Age, years	64.9 ± 11.2	64.9 ± 10.7	65.2 ± 10.8	65.5 ± 11.6	63.6 ± 10.4	68.2 ± 10.1	63.9 ± 11.0	65.1 ± 10.0	62.6 ± 11.8	62.2 ± 12.1	67.0 ± 13.0	66.7 ± 9.5
Women, %	29.6	26.8	30.8	28.0	23.0	21.5	20.8	38.4	28.5	26.4	30.7	45.3
PM_2.5_ WHO	38.1 ± 34.5	15.8 ± 5.7	20.7 ± 2.6	11.2*	10.1 ± 0.27	22.6 ± 7.4	10.6 ± 0.6	16.7 ± 4.4	20.5 ± 3.1	92.7 ± 31.6	34.1 ± 3.9	67.4 ± 14.7
PM_2.5_ Local	NA	15.0 ± 5.9	19.6 ± 4.1	10.6*	10.5 ± 0.5	20.3 ± 7.2	10.2 ± 1.0	NA	16.4 ± 3.4	NA	NA	86.3 ± 12.6
Smoker, %	16.1	17.9	20.5	23.2	14.6	14.6	20.0	25.5	17.3	10.3	20.0	12.3
Exercise, %	54.2	57.2	55.0	25.3	69.2	46.0	44.1	62.4	53.4	45	48.8	56.4
BMI, kg/m^2^	28.1 ± 34.5	28.6 ± 4.7	28.7 ± 4.2	28.3 ± 4.8	29.0 ± 5.0	26.9 ± 4.0	30.7 ± 5.5	29.5 ± 5.1	28.5 ± 4.5	30.3 ± 6.0	28.0 ± 4.0	25.0 ± 2.9
HTN, %	74.5	71.7	83.5	63.6	58.6	72.8	63.0	88.9	75.5	83.9	70.8	81.2
Diabetes, %	34.2	25.2	3.0	NA	17.9	30.2	14.8	24.0	26.7	76.1	35.8	45.8
Hyperlipidaemia, %	67.6	68.7	31.9	70.6	68.9	68.1	64.6	62.2	56.4	88.9	49.4	42.7
SBP, mmHg	131.1 ± 18.4	132.1 ± 19.3	133 ± 20.2	131 ± 19.4	134.2 ± 18.5	128.1 ± 18.9	121.8 ± 15.5	140.0 ± 14.5	136.5 ± 23.4	128.1 ± 17.0	131.5 ± 19.4	129.9 ± 13.2
DBP, mmHg	75.8 ± 10.9	76.7 ± 10.7	79.9 ± 11.6	76.0 ± 10.4	74.3 ± 10.0	75.4 ± 9.3	71.3 ± 8.1	79.9 ± 9.1	79.9 ± 12.7	71.3 ± 10.3	75.8 ± 12.7	77.5 ± 8.8
TC, mmol/L	4.2 ± 1.5	4.3 ± 1.7	4.7 ± 1.7	4.1 ± 0.9	4.0 ± 1.0	4.1 ± 2.2	3.9 ± 0.8	3.9 ± 1.8	4.9 ± 2.7	3.8 ± 1.0	4.3 ± 1.0	4.4 ± 1.0
LDL, mmol/L	2.4 ± 1.1	2.4 ± 1.2	2.8 ± 1.3	2.1 ± 0.8	2.1 ± 0.8	2.0 ± 1.0	1.9 ± 0.6	2.7 ± 1.3	3.5 ± 2.0	2.1 ± 0.8	2.7 ± 0.9	2.8 ± 0.9
HDL, mmol/L	1.1 ± 0.4	1.2 ± 0.4	1.1 ± 0.5	1.2 ± 0.4	1.2 ± 0.4	1.1 ± 0.4	1.2 ± 0.4	1.1 ± 0.4	1.1 ± 0.6	0.9 ± 0.3	1.2 ± 0.4	1.2 ± 0.4
Glucose, mmol/L	7.5 ± 1.5	6.1 ± 2.1	6.7 ± 2.6	6.4 ± 2.4	5.8 ± 1.4	6.1 ± 1.8	6.4 ± 2.6	5.3 ± 1.4	6.3 ± 2.7	7.8 ± 3.6	6.5 ± 2.5	6.2 ± 1.6

EU: Europe; NI: Northern Ireland; KSA: Saudi Arabia; SBP: systolic blood pressure; DBP: diastolic blood pressure; TC: total cholesterol; NA: not applicable. ‘Smoker’ was recorded as current smoker; ‘Exercise’ was recorded as adequate physical activities. Numeric variables are mean ± standard deviation and categorical variables are percentage. Units are years for age, kg/m^2^ for BMI, mmHg for SBP and DBP, mmol/L for TC, LDL, HDL, and glucose, and μg/m^3^ for PM_2.5_. * Only one centre from Denmark participated in SURF so standard deviation could not be provided.

### Associations between PM_2.5_ and cardiovascular risk factors

Appendix Figure C shows the crude association between PM_2.5_ and cardiovascular risk factors, indicating weak associations if any.

**Figure 1:**
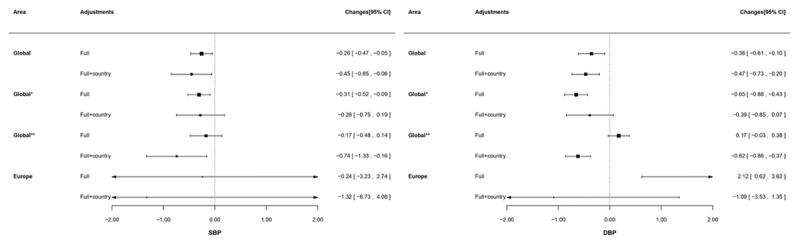
Changes (95% CI) in blood pressure increase in PM_2.5_ derived from World Health Organization. SBP: systolic blood pressure; DBP: diastolic blood pressure. All analyses were applied with generalized estimating equation model with centre clustered. ‘Full’ adjustment was sex, age, and risk factors (exercise, smoking status, and body mass index). ‘Full+country’ was sex, age, risk factors (exercise, smoking status, and body mass index), and country. Results are presented as changes in mmHg (95% CI). ‘Global*’ presented results are based on all participating countries except China; ‘Global**’ presented results are based on all participating countries except Saudi Arabia.

Globally, a 0.26 mmHg decrease in SBP per 10 μg/m^3^ increase in PM_2.5_ was observed (Figure 1). After controlling for country, the observed inverse association with SBP was slightly stronger but with wider confidence intervals (–0.45 mmHg; 95% CI: –0.85, –0.06). There were no statistically significant associations with SBP when the analysis was restricted to the European centers (1.32 mmHg; 95% CI: –6.73, 4.08).

Similar results were found for DBP: an increase of 10 μg/m^3^ in PM_2.5_ was associated with lower DBP (–0.36 mmHg; 95% CI: –0.10, 0.61) and the association tended to be stronger (–0.47 mmHg; –0.73, –0.20) after country adjustment on a global scale. On European level, a similar association between PM_2.5_ and DBP was observed which became non-significant after country adjustment.

**Figure 2:**
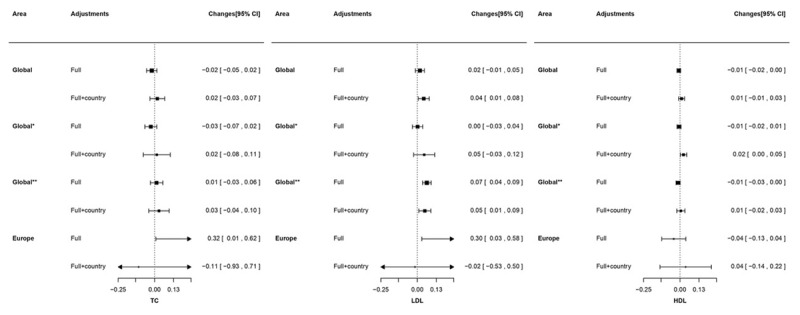
Changes (95% CI) in lipids (Total cholesterol, LDL-cholesterol, and HDL-cholesterol) increase in PM_2.5_ derived from World Health Organization. TC: total cholesterol; LDL: low-density lipoprotein cholesterol; HDL: high-density lipoprotein cholesterol. All analyses were applied with generalized estimating equation model with centre clustered. ‘Full’ adjustment was sex, age, and risk factors (exercise, smoking status, and body mass index). ‘Full+country’ was sex, age, risk factors (exercise, smoking status, and body mass index), and country. Results presented as changes in mmol/L (95% CI). ‘Global*’ presented results are based on all participating countries except China; ‘Global**’ presented results are based on all participating countries except Saudi Arabia. Globally, an increase of 10 μg/m^3^ PM_2.5_ was associated with an increased glucose level by 0.10 mmol/L (95% CI: 0.03 to 0.16). For Europe the increase in glucose was 0.30 mmol/L (95% CI: 0.06 to 0.53) (Figure 3). These associations, however, disappeared after adjustment for country.

Figure 2 shows the association between PM_2.5_ and lipid levels. Associations of PM_2.5_ with lipid levels were not statistically significant on a global scale. After controlling for country non-significant associations remained for TC and HDL; while, an increase of 10 μg/m^3^ in PM_2.5_ was associated with an increased LDL level (0.04 mmol/L, 95% CI: 0.01, 0.08). Weak positive associations of TC and LDL were observed among European participants (TC: 0.32 mmol/L; 95% CI: 0.01, 0.62; LDL: 0.30 mmol/L, 95% CI: 0.03, 0.58), which disappeared after adjustment for country. There was no significant association for HDL among European patients with or without adjustment for country.

**Figure 3:**
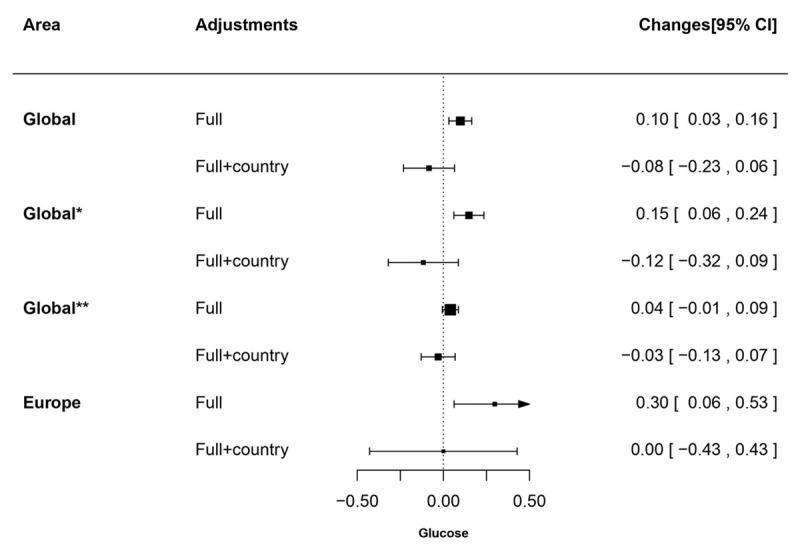
Changes (95% CI) in glucose increase in PM_2.5_ derived from World Health Organization. All analyses were applied with generalized estimating equation model with centre clustered. ‘Full’ adjustment was sex, age, and risk factors (exercise, smoking status, and body mass index). ‘Full+country’ was sex, age, risk factors (exercise, smoking status, and body mass index), and country. Results are presented as changes in mmol/L (95% CI). ‘Global*’ presented results are based on all participating countries except China; ‘Global**’ presented results are based on all participating countries except Saudi Arabia.

### Sensitivity analyses

Separate analyses with exclusion of China and Saudi Arabia (called as ‘global*’ and ‘global**’ in Figures 1, 2, 3) and with local PM_2.5_ exposure data (Appendix Table B) did not alter the main findings.

### Feasibility of data linkage

It is feasible to link existing cardiovascular risk factor data with PM_2.5_. During the linkage process, some limitations were identified in the various data sources. The air pollution data sources did not always contain data of the exact year of interest and thus we used the nearest by year. The SURF database did not contain individual addresses and hence we used the postal code of the hospital address of the patient as a proxy for the location of exposure to air pollution.

## Discussion

The analyses establish the technical feasibility of developing future data linkage studies but also point at challenges in their interpretation. In the current analysis, the long-term PM_2.5_ exposure from a consistent global exposure model was linked to individual data on routinely measured CVD risk factors from a large audit of 8,392 CHD patients from 71 centers in Europe, Asia, and the Middle East to explore potential association between air pollution and cardiovascular risk factors. Notably, taking country into account in the analyses materially affected the observed associations. While this adjustment may account for unmeasured confounding and lead to over adjustment.

### Associations between air pollution and cardiovascular risk factors

We observed an inverse association of PM_2.5_ with BP globally and among European participants after adjustment for country, which is in contrast with several previous studies that found positive associations between long-term exposures to PM_2.5_ and elevated BP [4,9,25,26]. Other studies found non-significant association [10]. For instance, findings from a national population-based study among 1024 elderly Taiwanese participants suggested that an interquartile increase in PM_2.5_ (48 μg/m^3^) is associated with 32.1 mmHg (95% CI 21.6–42.6) and 31.3 mmHg (95% CI 25.4–37.1) increases in SBP and DBP, respectively, after controlling age, sex, BMI, smoking, and drinking habitats [27]. A comprehensive meta-analysis among 113,926 patients from 15 European population-based cohort studies, ESCAPE, demonstrated inconsistent relationships between long-term exposure to modeled air pollutants including PM_2.5_ and BP in each cohort and the pooled results remained non-significant [10]. Studies on mechanisms have suggested that exposure to PM_2.5_ could instigate acute autonomic imbalance and then lead BP increases [4,5,25,28,29]. However, our study was conducted in CHD patients who all received cardiovascular medications to control potential risk factors. Consequently, we measured the potential impact of air pollution beyond medical treatment. Future linkage studies would need to include both treated and untreated patients to better investigate the association between air pollution and CHD risk factors.

Some previous evidence suggested that PM_2.5_ may affect lipid levels but the quantity and quality of these studies is still limited and results are not fully consistent [27,30,31]. A large cross-sectional study with 39,863 healthy participants in Denmark demonstrated that the interquartile range (11.3 μg/m^3^) of PM_2.5_ was associated with a higher level of TC (0.78 mg/dl; 95% CI: 0.22–1.34) [31]. An animal study also indicated that mice exposed to PM_2.5_ had significantly higher levels of TC and LDL than those exposed to filtered air [30]. However, effect estimates are typically small and may have little clinical implications.

We observed direct associations of PM_2.5_ with glucose in both global and European analyses, although these associations attenuated after country adjustment. These findings are in line with previous studies [32,33]. A cross-sectional study based on Chinese populations reported that both elevated glucose levels and increased type II diabetes prevalence are significantly associated with increased PM_2.5_ [34]. A review from 21 published studies reported concentrations of PM_2.5_ to be associated with increased insulin resistance and higher rates of type II diabetes [32]. Mechanisms suggested to link glucose metabolism to PM_2.5_ with endothelial dysfunction, endoplasmic reticulum stress, insulin signaling abnormalities, and systematic inflammation [5,12,33,34]. Differences in the study characteristics, population characteristics, and exposure duration in different geographic research areas may contribute to the discrepancies in these findings.

### Feasibility and challenges

The current study has piloted feasibility to add air pollution exposure using a coherent methodology to the rich database of clinical observations on cardiovascular risk factors from SURF. Data linkage is a robust, valuable and cost-effective research tool for combining individual level data from different sources for maximizing use of these existing database and increasing amounts of data that are being produced in order to: 1) address clinical research questions that require large sample sizes, detailed data on hard-to-reach population, or specified measurements by using a single dataset; 2) generate evidence with a high level of external validity and applicability; 3) reduce participant burden and avoid duplication of effort [16,35,36,37]. This study facilitates data-linkage possibilities to investigate the impact of air pollution on CHD on a global scale, which is important clinical practice. A recent study demonstrated that the contribution of air pollution to CVD is comparable to that of smoking [38]. Such efficient and cost-effective methods enable all healthcare providers to enrich clinical data to investigate novel health-related research questions.

### Limitations

There are several limitations in this study. SURF records CHD management in daily practice. Unlike other epidemiological studies, physical and laboratory measurements are not standardized. Some potential confounders were not available and thus could not be adjusted for. In addition, SURF collected anonymous data and thus only participating center’s locations at aggregated level were linked to air pollution data instead of individual level, which may not reflect actual exposure at the individual level. However, most routine cardiology visits were conducted in local hospitals with the distance between home and clinic generally being less than 10 km as confirmed by SURF national collaborators for 80%–90% of their patients had their residence near hospitals. The lack of individual addresses resulted in that only PM_2.5_ concentrations were assigned to each center, as PM_2.5_ is a regionally varying pollutant with limited small-scale spatial variation [21]. Finally, data on other spatially-correlated air pollution factors, such as traffic noise, greenness, and urbanity, were not taken into account and thus not adjusted for, potentially under- or overestimating results.

Furthermore, the air pollution data from WHO database was not available for 2013, which was our year of interest because this coincides with the year of observation for the SURF study. In sensitivity analysis, we further analyzed association with PM_2.5_ exposure data provided by local resource from 2013 and found that the results are broadly similar. Annual average concentrations may vary from year to year due to variations in weather, but the spatial contrasts in air pollution are typically stable over years and as such it may only have limitedly impacted our findings [22,23,24].

While most epidemiological studies of air pollution are based upon more individual exposure assessment, our approach does not invalidate the epidemiological study. First, the selected pollutant PM_2.5_ mostly varies on a regional scale with limited local variability. In a large monitoring study across Europe, we observed that 81% of the variance was due to between study area variability [21]. Second, people do not spend only time at their residence but in a wider neighborhood, arguing for exposure assessment at a larger scale. Third, the error made by assigning an area-level estimate may lead to Berkson rather than classical error which would not bias air pollution effect estimates but only increase imprecision [39]. Fourth, if the contrast in exposure is large between study areas, assigning an area-level value may be acceptable. Recent studies have applied this approach in settings with large exposure contrasts [40]. Therefore, we would like to clarify that current study was an attempt to use clinical audit data to investigate more health related research questions beyond cardiovascular risk factor management. Further research is needed to validate current findings due to these methodological limitations.

### Further direction

We hope that our findings may stimulate linkage studies on cardiovascular risk and disease in primary prevention settings in which relationships may be stronger and the findings less likely to be confounded by medication. Even though clinicians may not be able to change patients’ living environment they should become more aware of the hazards of air pollution and take it into account in their risk assessment and recommendations, such as promoting exercise in less polluted areas.

## Conclusions

The current study has demonstrated the feasibility of data. The approach exemplifies the opportunity to assess the impact of the environment on cardiovascular risk factors across large geographic areas. We noted that estimates were highly sensitive to adjustment for country. After country adjustment, PM_2.5_ levels are marginally associated with increases in LDL cholesterol and decreases in BP. The implication is that similar global studies should aim at multiple centers per country with sufficient within country exposure contrast to balance any effects of over adjustment.

## Data Accessibility Statement

The data that support the findings of this study are available from the SURF project, which have been published previously. The references have been included in the current study.

## Additional Files

The additional files for this article can be found as follows:

10.5334/gh.877.s1 Appendix Table A. Missing data in SURF.

10.5334/gh.877.s2 Appendix Table B. Associations between CVD risk factors and PM2.5 retrieved from WHO, airbase for European countries and local government database for China.

10.5334/gh.877.s3 Appendix Table C. Risk factors, stratified by centres.

10.5334/gh.877.s4 Appendix Figure A. PM_2.5_ distribution.

10.5334/gh.877.s5 Appendix Figure B. Variations of individual outcome variable by country.

10.5334/gh.877.s6 Appendix Figure C. Crude association between PM_2.5_ and cardiovascular risk factors.
