# Supplementary material for: A Global Analysis of Associations between Fine Particle Air Pollution and Cardiovascular Risk Factors: Feasibility Study on Data Linkage
Source: Glob Heart. 2020 Aug 6;15(1):53. doi: 10.5334/gh.877 (PMC7427684; doi:10.5334/gh.877)
Supplement: Appendix Table A. — Missing data in SURF. [file gh-15-1-877-s1.pdf]

Supplements

Table A. Missing data in SURF

Variables	No. of Missing	Imputed in analysis
Country	0	No
Centre	0	No
Age	0	No
Sex	0	No
PM2.5	0	No
Smoking	84	Yes
Systolic blood pressure (SBP)	93	Yes
Diastolic blood pressure (DBP)	95	Yes
Exercise	127	Yes
Weight	227	Yes
Height	357	Yes
Total cholesterol (TC)	760	Yes
Low-density lipoprotein (LDL)	1254	Yes
High-density lipoprotein (HDL)	1139	Yes
Glucose	1093	Yes
