# Supplementary material for: A Global Analysis of Associations between Fine Particle Air Pollution and Cardiovascular Risk Factors: Feasibility Study on Data Linkage
Source: Glob Heart. 2020 Aug 6;15(1):53. doi: 10.5334/gh.877 (PMC7427684; doi:10.5334/gh.877)
Supplement: Appendix Table B. — Associations between CVD risk factors and PM2.5 retrieved from WHO, airbase for European countries and local government database for China. [file gh-15-1-877-s2.pdf]

Table B. Associations between CVD risk factors and PM2.5 retrieved from WHO, airbase for European countries and local government database for China

		SBP (mmHg)	DBP (mmHg)	TC (mmol/L)	LDL (mmol/L)	HDL (mmol/L)	Glucose (mmol/L)
Croatia	WHO	5.4 (-2.0, 12.8)	-0.14 (-1.0, 0.7)	0.5 (-0.1, 1.0)	0.6 (0.3, 0.9)	0.08 (-0.2, 0.3)	-0.1 (-1.0, 0.7)
	Airbase	3.6 (-0.5, 7.7)	1.5 (-1.0, 4.0)	-0.02 (-0.1, 0.07)	0.1 (-0.4, 0.7)	-0.2 (-0.5, 0.04)	0.1 (-0.6, 0.8)
Ireland	WHO	59.5 (14.2, 104.8)	-3.1 (-10.5, 4.4)	-2.2 (-5.6, 1.2)	-2.0 (-4.3, 0.3)	0.3 (-0.1, 0.8)	-3.1 (-10.5, 4.4)
	Airbase	24.8 (6.6, 43.0)	3.3 (-6.0, 12.6)	-0.03 (-1.3, 0.7)	-0.07 (-1.4, 0.05)	0.07 (-0.1, 0.3)	-3.7 (-5.6, -1.9)
Italy	WHO	-4.9 (-14.4, 4.6)	0.15 (-0.3, 0.6)	-0.5 (-1.9, 0.9)	-0.1 (-0.5, 0.2)	0.1 (-0.4, 0.3)	0.2 (-0.3, 0.6)
	Airbase	-2.7 (-8.1, 2.7)	-0.7 (-3.5, 2.1)	-0.3 (-0.9, 0.3)	-0.05 (-0.3, 0.2)	0.05 (-0.07, 0.2)	0.3 (0.02, 0.5)
Romania	WHO	-6.1 (-30.9, 18.7)	-0.6 (-2.5, 1.2)	-2.0 (-5.8, 1.8)	-1.4 (-4.0, 1.2)	-0.3 (-1.1, 0.6)	-0.6 (-2.5, 1.2)
	Airbase	5.6 (-6.0, 17.1)	-0.2 (-7.5, 4.2)	-3.2 (-8.3, 1.8)	-1.9 (-5.3, 1.5)	-0.7 (-1.7, 0.2)	-1.7 (-3.8, 0.4)
Europe	WHO	-0.2 (-3.2, 2.7)	2.1 (0.6, 3.6)	0.3 (0.009, 0.6)	0.3 (0.03, 0.6)	-0.04 (-0.1, 0.04)	0.3 (0.06, 0.5)
	Airbase	-1.0 (-4.2, 2.2)	1.4 (-0.2, 3.0)	-0.07 (-0.4, 0.3)	0.05 (-0.2, 0.3)	-0.08 (-0.2, 0.02)	0.2 (-0.09, 0.5)
China	WHO	-0.3 (-1.0, 0.4)	-0.7 (-1.2, -0.3)	-0.2 (-0.1, 0.7)	0.01 (-0.04, 0.06)	-0.1 (-0.2, -0.06)	-0.3 (-0.4, -0.2)
	Local	-0.04 (-1.4,0.5)	-0.7(-1.3, -0.09)	-0.05(-0.2,0.05)	-0.03(-0.1,0.05)	-0.1(-0.2, -0.002)	-0.3 (-0.5, -0.09)

SBP: systolic blood pressure; DBP: diastolic blood pressure; TC: total cholesterol.

Associations were presented as changes (95% confidence interval) with 10 μg/m^3 increased in PM2.5 from model with gender, age, smoking status (current smoker, ex-smoker, and never smoke), physical activity (Less than recommended exercise, moderate exercise, and more than recommended exercise), and body mass index (under weight, normal weight, overweight, and obese) adjusted.
