# Supplementary material for: A Global Analysis of Associations between Fine Particle Air Pollution and Cardiovascular Risk Factors: Feasibility Study on Data Linkage
Source: Glob Heart. 2020 Aug 6;15(1):53. doi: 10.5334/gh.877 (PMC7427684; doi:10.5334/gh.877)
Supplement: Appendix Table C. — Risk factors, stratified by centres. [file gh-15-1-877-s3.pdf]

Table C. Risk factors, stratified by centres

	SBP (mmHg)	DBP (mmHg)	TC (mmol/L)	LDL (mmol/L)	HDL (mmol/L)	Glucose (mmol/L)
Croatia 1	127.6±18.1	76.7±9.1	5.2±1.5	2.5±1.2	1.1±0.4	7.4±3.1
Croatia 2	132.2±15.0	80.6±8.7	4.5±1.1	2.7±1.0	1.3±0.4	6.6±2.0
Croatia 3	141.0±26.5	79.6±14.7	5.2 ±1.5	3.3±1.2	1.1±0.4	8.0±3.5
Croatia 4	131.4±20.1	79.4±10.6	5.2±1.0	3.0±1.2	1.3±0.4	6.3±1.6
Croatia 5	131.8±18.5	77.9±10.5	4.3±1.3	2.5 ±1.1	1.1±0.3	6.6±2.4
Croatia 6	137.2±21.8	82.4±12.5	5.2±1.4	3.2±1.2	1.2±0.4	7.1±2.5
Croatia 7	131.8±19.5	79.1±10.8	4.7±1.5	2.9±1.2	1.2±0.4	6.4±2.0
Croatia 8	128.2±22.7	77.3±13.9	3.5±2.8	2.1±1.7	1.6±0.5	5.8±3.2
Croatia 9	136.5±21.6	80.9±12.2	4.5±1.8	2.6±1.5	0.9±0.5	7.2±3.7
Ireland 1	128.9±15.8	73.2±9.3	3.6±0.8	1.8±0.6	1.2±0.3	5.7±1.3
Ireland 2	126.8±16.8	74.1±8.0	3.9±1.0	2.0±0.8	1.2±0.4	5.4±1.1
Ireland 3	134.4±17.2	75.3±9.2	3.7±0.8	1.9±0.7	1.2±0.3	5.6±1.0
Ireland 4	130.0±14.7	72.1±9.3	3.5±0.8	1.8±0.7	1.3±0.3	5.4±0.6
Ireland 5	134.7±14.8	74.2±12.5	4.5±1.2	2.5±1.1	1.2±0.3	5.6±1.0
Ireland 6	137.1±24.6	78.1±10.5	3.8±1.1	1.9±0.8	1.2±0.4	5.7±1.1
Ireland 7	123.9±12.5	74.1±10.1	4.1±1.2	2.3±1.0	1.2±0.4	6.8±2.8
Ireland 8	137.3±19.0	74.7±9.8	4.2±1.1	2.3±0.9	1.3±0.4	5.6±1.1
Ireland 9	126.8±15.4	75.3±14.0	3.9±0.7	2.3±0.6	1.1±0.2	5.7±0.7
Ireland 10	136.2±18.6	74.0±10.0	4.1±1.1	2.2±0.8	1.2±0.4	5.9±1.6
Ireland 11	139.1±28.2	76.4±14.1	4.1±1.0	2.0±0.8	1.2±0.2	5.4±0.7
Italy 1	132.4±12.2	79.0±7.7	4.3±1.1	2.5±0.8	1.1±0.3	NA
Italy 2	140.7±21.2	82.6±10.7	4.3±1.0	2.3±0.7	1.3±0.4	5.8±1.4
Italy 3	121.2±13.5	68.8±6.8	3.3±1.6	1.8±1.2	0.8±0.2	5.6±1.3
Italy 4	144.7±24.2	80.4±10.8	4.1±1.1	2.2±0.8	1.1±0.3	6.2±1.2
Italy 5	121.4±8.8	75.5±6.4	4.1±1.0	2.2±0.7	1.3±0.5	6.0±1.5
Italy 6	125.2±13.2	70.9±8.1	3.9±0.9	2.1±0.7	1.1±0.3	5.6±1.4
Italy 7	132.0±19.6	76.6±9.3	3.6±0.9	2.1±1.2	1.5±0.7	6.5±1.3
Italy 8	119.8±13.0	75 ±6.3	3.5±0.8	2.1±0.5	NA	7.4±3.4
Italy 9	145.4±26.8	79.9±8.2	4.2±1.0	2.0±0.6	1.2±0.4	6.1±1.4
Italy 10	124.7±16.1	78.8±6.0	3.8±0.9	2.1±0.7	1.1±0.3	6.3±1.9
Italy 11	124.3±13.9	75.1±7.6	8.4±6.0	3.2±1.5	1.1±0.2	6.2±1.9
Italy 12	122.6±20.6	75.0±8.9	4.3±1.6	2.3±0.8	1.1±0.3	6.3±1.9
Italy 13	129.8±17.1	74.8±10.2	4.3±0.8	2.4±0.6	1.2±0.3	6.2±1.4
Italy 14	132.5±18.0	80.2±9.1	4.2±0.8	3.4±1.6	1.3±0.4	6.3±1.0
Denmark 1	131.0±19.4	76.0±10.4	4.1±0.9	2.1±0.8	1.2±0.4	6.4±2.4
UK 1	123.8±15.3	72.5±7.2	3.9±0.8	1.9±0.6	1.1±0.3	6.7±3.3
UK 2	118.0±15.1	68.9±8.9	3.8±0.7	1.9±0.7	1.2±0.4	5.9±1.2
KSA 1	129.2±17.5	70.1±10.5	4.0±1.0	2.3±0.8	1.0±0.3	8.1±3.9
KSA 2	127.2±21.1	68.0±10.9	4.5±1.5	2.7±1.2	1.3±0.6	6.9±4.1
KSA 3	135.2±20.1	75.2±11.2	4.2±1.1	2.1±0.7	1.1±0.2	9.5±4.8
KSA 4	128.2±18.9	71.6±10.6	3.9±1.0	2.2±0.7	1.0±0.3	7.9±3.2
Romania 1	133.2±21.5	80.4 ±12.4	4.4±1.0	2.6±1.0	1.2±0.3	6.7±2.4
Romania 2	127.0±23.6	75.3±7.2	2.2±1.6	NA	0.5±0.5	4.2±1.4
Romania 3	136.7±26.2	81.3±13.7	4.5±1.4	NA	NA	6.6±1.7
Romania 4	133.1±18.1	77.2±10.1	3.7±1.8	2.0±1.3	0.9±0.4	5.7±2.2
Romania 5	145.4±28.7	80.2±13.0	5.1±1.2	3.4±1.1	1.2±0.5	6.6±2.2
Romania 6	128.3±28.7	77.4±13.8	9.7±2.1	6.3±1.1	1.9±0.4	8.2±3.9

	SBP (mmHg)	DBP (mmHg)	TC (mmol/L)	LDL (mmol/L)	HDL (mmol/L)	Glucose (mmol/L)
Romania 7	157.0±22.7	91.7±14.1	5.0±3.0	3.0±1.0	1.0±0.2	6.6±2.1
Russia 1	132.1±15.1	82.7±9.8	5.8±1.2	3.1±0.9	1.2±0.4	6.1±0.8
Russia 2	130.5±15.4	80.3±8.4	5.5±0.3	1.8±0.3	1.3±0.1	3.7±1.3
Russia 3	129.6±10.7	78.5±6.1	5.7±1.7	3.8±1.4	1.1±0.3	5.4±0.9
Russia 4	128.5±17.0	80.3±10.2	4.4±0.9	1.8±0.4	1.1±0.4	5.2±1.2
Russia 5	124.1±11.4	78.2±6.5	4.6±0.9	3.2±0.6	1.0±0.2	5.9±1.1
Russia 6	132.0±14.2	80.3±10.9	5.1±0.9	3.1±0.9	1.2±0.2	5.6±1.2
Russia 7	129.0±13.4	78.9±7.5	5.0±1.1	3.8±1.7	1.3±0.2	5.6±0.9
Russia 8	128.6±16.5	80.4±12.2	5.1±1.1	2.7±1.3	1.3±0.5	5.7±1.4
Taiwan 1	136.6±21.9	76.2±14.4	4.4±1.0	2.7±0.9	1.2±0.4	7.2±3.4
Taiwan 2	130.2±18.0	74.0±11.9	4.5±1.0	2.7±0.8	1.1±0.4	6.3±1.8
Taiwan 3	127.5±17.0	75.7±11.5	4.2±0.9	2.6±0.9	1.2±0.3	6.0±2.0
Taiwan 4	135.9±18.8	80.6±12.7	4.2±1.0	2.7±1.0	1.1±0.3	6.6±2.2
China 1	129.9±12.9	78.0±7.6	4.4±1.1	2.9±1.0	1.0±0.3	6.1±2.1
China 2	129.3±12.6	77.6±8.1	4.4±1.1	2.8±1.0	1.0±0.2	5.6±1.2
China 3	131.0±13.5	79.4±8.2	4.3±0.9	2.8±0.9	1.0±0.3	6.0±1.7
China 4	130.0±13.6	76.4±8.8	4.4±1.0	2.8±0.9	1.0±0.2	5.7±1.3
China 5	127.1±14.4	74.0±8.8	4.3±1.0	2.8±0.9	1.1±0.3	5.9±1.5
China 6	127.0±12.0	76.1±8.9	3.9±1.0	2.3±0.9	0.9±0.3	5.9±1.8
China 7	128.8±12.6	78.5±7.0	4.0±0.9	2.6±0.8	1.0±0.2	5.7±1.4
China 8	130.2±12.0	79.0±8.9	4.4±0.9	2.8±0.8	1.2±0.4	5.6±1.3
China 9	133.6±21.4	80.2±14.7	3.9±1.1	2.3±1.0	1.0±0.2	5.7±1.7
China 10	132.3±14.2	78.9±9.3	4.6±0.9	2.8±0.9	1.6±0.4	7.1±1.7
China 11	129.3±12.3	77.2±9.1	4.5±0.9	2.9±0.8	1.5±0.3	6.7±1.3

SBP: systolic blood pressure; DBP: diastolic blood pressure; TC: total cholesterol; LDL: low-density lipoprotein cholesterol; HDL: high-density lipoprotein cholesterol

Unit: mmHg for SBP and DBP; mmol/L for TC, LDL, HDL, and glucose
