# Supplementary figures and images for: A Global Analysis of Associations between Fine Particle Air Pollution and Cardiovascular Risk Factors: Feasibility Study on Data Linkage
Source: Glob Heart. 2020 Aug 6;15(1):53. doi: 10.5334/gh.877 (PMC7427684; doi:10.5334/gh.877)

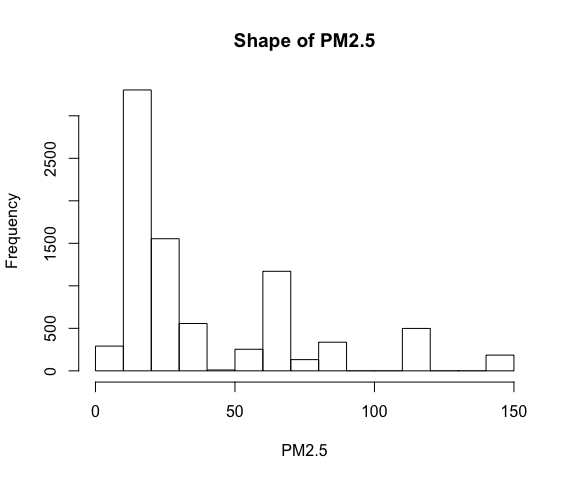

Supplement: Appendix Figure A. — PM2.5 distribution. [file gh-15-1-877-s4.png]

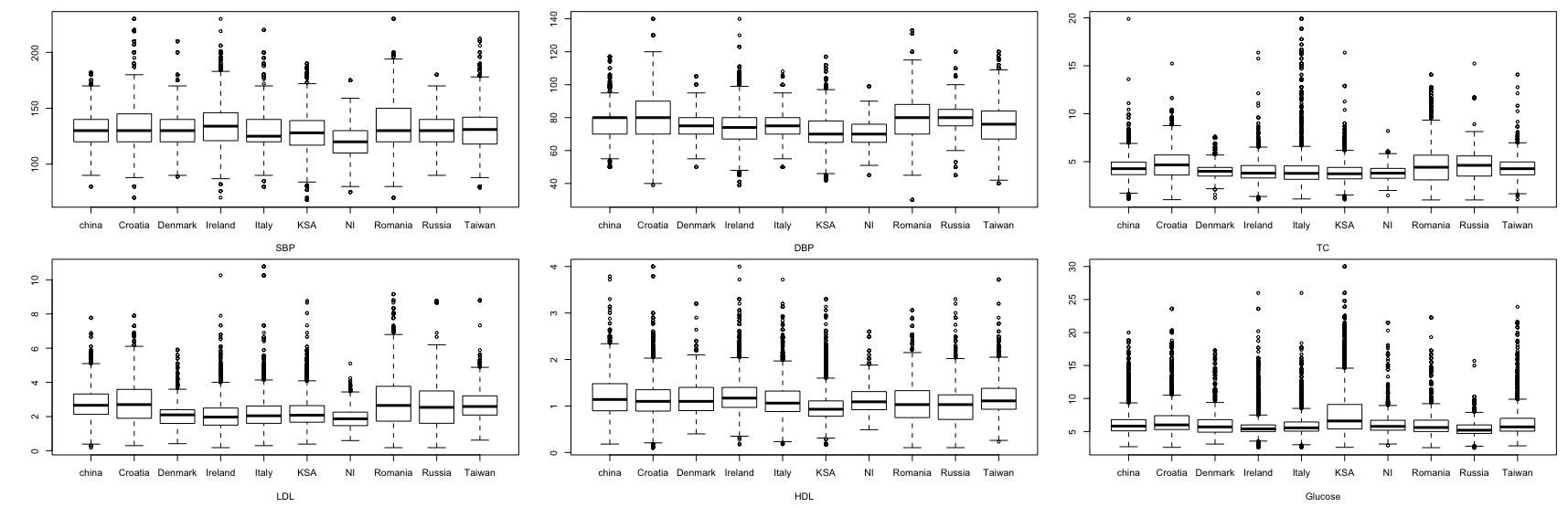

Supplement: Appendix Figure B. — Variations of individual outcome variable by country. [file gh-15-1-877-s5.png]

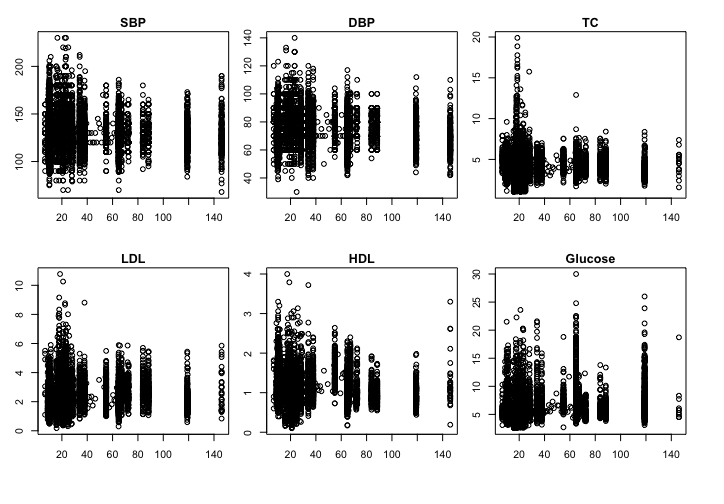

Supplement: Appendix Figure C. — Crude association between PM2.5 and cardiovascular risk factors. [file gh-15-1-877-s6.png]
